# Supplementary material for: An analysis of deficiencies in the ethics committee data of certain interventional trials registered with the Clinical Trials Registry–India
Source: PLOS Glob Public Health. 2022 Oct 24;2(10):e0000617. doi: 10.1371/journal.pgph.0000617 (PMC10021301; doi:10.1371/journal.pgph.0000617)
Supplement: S1 File — (PDF) [file pgph.0000617.s001.pdf]

S1 File. A sample CTRI record.

[...ck Here to Create PDF for Current Dataset of Trial]

**CTRI Number**	**CTRI/2020/09/027903** [Registered on: 18/09/2020] **Trial Registered Prospectively**
**Last Modified On:**	05/01/2021
**Post Graduate Thesis**	No
**Type of Trial**	Interventional
**Type of Study**	Biological
**Study Design**	Other
**Public Title of Study**	Testing the efficacy and safety of a blood product COVID-19 Hyper-Immuneglobulin (Human) Solution in Participants with Active COVID-19
**Scientific Title of Study**	A Prospective, Open-Label, Two-Arm, Parallel-Group, Randomized, Controlled, Multi-Centric Trial for Evaluation of Efficacy and Safety of COVID-19 Hyper-Immuneglobulin (Human) Solution in Participants with Active COVID-19
**Trial Acronym**	

Secondary IDs if Any

Secondary ID	Identifier
0279-20, Version 2.0, Dated: 28-Aug-2020	Protocol Number

Details of Principal Investigator or overall Trial Coordinator (multi-center study)

**Name**	Mr Prashant Modi
**Designation**	Sr. General Manager
**Affiliation**	Lambda Therapeutic Research Ltd
**Address**	Lambda House, Department of Project Management & Regulatory Affairs, Plot No. 38, Survey No. 388 Near Silver Oak Club, S. G. Highway,Gota<br><br>Ahmadabad<br>GUJARAT<br>382481<br>India
**Phone**	917940202375
**Fax**	07940202021
**Email**	prashantmodi@lambda-cro.com

Details of Contact Person Scientific Query

Name	Dr Naman Shah
**Designation**	General Manager
**Affiliation**	Lambda Therapeutic Research Ltd
**Address**	Lambda House, Department of CTM Medical Services, Plot No. 38, Survey No. 388 Near Silver Oak Club, S. G. Highway,Gota<br><br>Ahmadabad<br>GUJARAT<br>382481<br>India
**Phone**	07940202389
**Fax**	07940202021
**Email**	namanshah@lambda-cro.com

Details of Contact Person Public Query

**Name**	Mr Prashant Modi
**Designation**	Sr. General Manager
**Affiliation**	Lambda Therapeutic Research Ltd
**Address**	Lambda House, Department of Project Management & Regulatory Affairs, Plot No. 38, Survey No. 388 Near Silver Oak Club, S. G. Highway,Gota<br><br>Ahmadabad<br>GUJARAT<br>382481<br>India
**Phone**	917940202375
**Fax**	07940202021
**Email**	prashantmodi@lambda-cro.com

Source of Monetary or Material Support

Intas Pharmaceuticals Limited, Corporate House, Nr. Sola Bridge, S.G. Highway, Thaltej, Ahmedabad– 380054, Gujarat, India.

Primary Sponsor

**Name**	Intas Pharmaceuticals Limited
**Address**	Corporate House, Nr. Sola Bridge, S.G. Highway, Thaltej, Ahmedabad– 380054, Gujarat, India
**Type of Sponsor**	Pharmaceutical industry-Indian

Details of Secondary Sponsor

Name	Address
NIL	NIL

Countries of Recruitment

India

Sites of Study
Modification(s)

No of Sites = **10**

Name of Principal Investigator	Name of Site	Site Address	Phone/Fax/Email
Dr Ravi Nagarajaiah	Adichunchanagiri Hospital & Research Centre	Department of Clinical Research, Room No.NA, B G Nagara, Nagamangala Taluk - 571448<br>Mandya<br>KARNATAKA	9448323893<br><br>ravibn972@yahoo.com
Dr Amit Patel	CIMS Hospital Pvt Ltd.	Department of Clinical Research, Room No.NA, Opp. Panchamrut Bunglows, Nr. Shukan Mall, Off Science City Road, Sola- 380060<br>Ahmadabad<br>GUJARAT	9824310150<br><br>amit.patel@cimshospital.org
Dr Deepak Namjoshi	Criticare Hospital	J Plot No 516, Besides SBI, Teli Gali, Andheri East, Mumbai - 400069<br>Mumbai<br>MAHARASHTRA	9320247247<br><br>investigatorresearch@rediffmail.com
Dr Chirag Rathod	GMERS Medical College & Hospital	Department of Clinical Research, Room No.NA, Gotri Road, Gotri - 390021<br>Vadodara<br>GUJARAT	9164636137<br><br>GMERS.trials@spearmind.com
Dr Rajesh Gosavi	Government Medical College & Hospital, Dr. Rajesh Gosavi	Department of Clinical Research, Room No.NA, Department of Radiation Therapy & Oncology, Government Medical College & Hospital, Medical College Square Road- 440003<br>Nagpur<br>MAHARASHTRA	9890225111<br><br>GOSAVIRV@hotmail.com
Dr Vinay Kumar	GSVM Medical College	Department of Clinical Research, Room No. NA, Post Graduate Department of Medicine, Swaroop Nagar -208002<br>Kanpur Nagar<br>UTTAR PRADESH	8726555577<br><br>dr.vinayksachan@gmail.com
Dr Amit Shah	Metas Adventis Hospital	Department of Clinical Research, Room No. 13-B, Nondh No 0363 To 0365, RS No 21, Opp.Chowpati Road, Athwalines CITY - 395001<br>Surat<br>GUJARAT	9824483868<br><br>dramitsshah@gmail.com
Dr Ambuj Garg	Sir Ganga Ram Hospital	Department of Clinical Research, Room No. NA, Sir Ganga Ram Hospital Marg, Rajinder Nagar, New-Delhi - 110060<br>New Delhi<br>DELHI	9810092313<br><br>drambujgarg@gmail.com
Dr K Vengadakrishnan	Sri Ramachandra Institute of Higher Education and Research(Deemed to be University)	Department of Clinical Research, Room No. 1, Ramachandra Nagar, Porur, Chennai - 600116<br>Chennai<br>TAMIL NADU	9840131997<br><br>drkvk1975@gmail.com
Dr Abhay Vispute	SRV Hospital	Dr. Mandikini Parihar Marg, Opposite Lokmanya Tilak Terminus, TilakNagar Chembur- 400089<br>Mumbai<br>MAHARASHTRA	9223247247<br><br>surgician@gmail.com

Details of Ethics Committee
Modification(s)

No of Ethics Committees= **10**

Name of Committee	Approval Status
Ethics Committee - Metas Adventist Hospital, Dr. Amit Shah	Approved
Ethics Committee of Care Institute of Medical Sciences, Dr. Amit Patel	Approved
Ethics Committee, GSVM Medical College, Dr. Vinay Kumar	Approved
Institutional Ethics Commitee Department of Pharmacology, Government Medical College, Dr. Rajesh Gosavi	Approved
Institutional Ethics Committee Adichunchanagiri University Adichunchanagiri Hospital & Research Centre, Dr Ravi Nagarajaiah	Approved
Institutional Ethics Committee - SRIHER, Dr. K Vengadakrishnan	Approved
Institutional Ethics Committee Centre for Research SRV Hospital(IECCRSRV), Dr Abhay Vispute	Approved
Institutional Ethics Committee Centre for Research SRV Hospital(IECCRSRV), Dr Deepak Namjoshi	Approved
Institutional Human Ethics Committee, GMERS Medical College & Hospital, Dr. Chirag Rathod	Approved
Sir Ganga Ram Hospital Ethics Committee, Dr. Ambuj Garg	Approved

Regulatory Clearance Status from DCGI

Status
Approved/Obtained

Health Condition / Problems Studied

Health Type	Condition
Patients	(1) **ICD-10 Condition:** B972‖Coronavirus as the cause of diseases classified elsewhere,

Intervention / Comparator Agent

Type	Name	Details
Intervention	COVID-19 Hyper-Immuneglobulin (Human) solution	Manufacturer- Intas Pharmaceuticals Limited; Dosage Level(s)- 30 mL as an intravenous injection on day 1 & 2 at the rate of not more than 0.5mL/kg/h; Route of Administration- Intravenous injection
Comparator Agent	Standard of care	Standard of care is the treatment algorithm/modalities to be given at discretion of the investigator as defined in the latest Guidelines on Clinical Management of COVID-19 issued by Ministry of Health and Family Welfare, Government of India

Inclusion Criteria

**Age From**	18.00 Year(s)
**Age To**	65.00 Year(s)
**Gender**	Both

Details

1
Participant and/or legally acceptable representative must sign an ICF to participate in the study indicating that the participant understands the purpose of, and procedures required for the study as described in this protocol and is willing to and will be able to adhere to requirement of the protocol.
2
Participant must be 18 to 65 years of age (both inclusive), at the time of signing the informed consent.
3
Participants must have documented laboratory-confirmed SARS-CoV-2 infection as determined by reverse transcription- polymerase chain reaction (RT-PCR) in any specimen, within less than 72 hours prior to randomization;
4
Participants with moderate or severe active COVID-19 (Clinical Management of COVID-19 Guidelines of MOHFW) at screening and baseline defined as
a. Radiological evidence of pulmonary infiltrates or Clinical features such as dyspnea and/or hypoxia, fever, cough, AND
b. SpO2 of less than 94 % on room air AND
c. Respiratory rate of greater than or equal to 24 per minute
5
A female participant is eligible to participate if she is not pregnant or breastfeeding, and at least one of the following conditions applies:
a. Is not a woman of childbearing potential (WOCBP)
OR
b. Is a WOCBP and using an acceptable contraceptive method as described in Appendix 10.4 during the intervention period and at a minimum 30 days until after the last dose of study intervention. The investigator should evaluate the effectiveness of the contraceptive method in relationship to the first dose of study intervention.
c. A WOCBP must have a negative highly sensitive pregnancy test [serum] within 4 days before the first dose of study intervention.
d. Additional requirements for pregnancy testing during and after study intervention are in Appendix 10.2
e. The investigator is responsible for review of medical history, menstrual history, and recent sexual activity to decrease the risk for inclusion of a woman with an early undetected pregnancy.
6
Male participants are eligible to participate if they agree to the following during the intervention period and for at least 90 days after the last dose of study intervention:
a. Must agree not to donate sperm for the purpose of reproduction
PLUS
b. Must agree to use contraception /barrier as detailed below
i. a male participant must wear a condom when engaging in any activity that allows for passage of ejaculate to another person
ii. Should also be advised of the benefit for a female partner to use a highly effective method of contraception described in Appendix 10.4 as a condom may break or leak when having sexual intercourse with a woman of childbearing potential who is not currently pregnant

ExclusionCriteria

Details

1
Participant requiring invasive ventilation or having hemodynamic instability (MOHFW guideline) or multiple organ dysfunction/failure or evidence of bacterial superinfection (as defined by Procalcitonin level greater than or equal to 0.5 μg/L or other applicable diagnostic parameters as per standard medical care) as per the independent clinical judgment of the Investigator at screening and /or baseline.
2
Documented medical history of known allergies, hypersensitivity, or intolerance to intravenous immunoglobulin or other injectable form of IgG or blood products.
3
Documented medical history of known IgA deficiency.
4
Participants with a lifetime history of at least one thrombotic event including deep vein thrombosis, cerebrovascular accident, pulmonary embolism, transient ischemic attacks, or myocardial infarction.
5
Participants who have received any blood products within 30 days prior to randomization.
6
Participant with more than 5 days of COVID-19 specific hospitalization prior to the first administration of treatment at baseline.
7
Participants who have more than 10 days between the onset of symptoms and the day of first administration of treatment at baseline.
8
Pregnant or breastfeeding female participants.
9
Currently receiving renal replacement therapy/dialysis OR Creatinine clearance less than 50 mL/min using the Cockcroft-Gault formula.
10
Documented medical history of hepatitis B surface antigen (HBsAg) or hepatitis C antibody (anti-HCV) positive, or other clinically active liver disease, or tests positive for HBsAg or anti-HCV at Screening.
11
Documented medical history of human immunodeficiency virus (HIV) antibody positive, or tests positive for HIV at Screening.
12
Currently receiving or has received in the last 14 days, experimental immune modulators, and/or monoclonal antibody therapies
13
Confirmed diagnosis of bacterial pneumonia or other active/uncontrolled fungal or viral infections at screening/baseline
14
Participants who have received organ transplantation or major surgery in the past 6 months.
15
Participants whose ALT/AST levels are 5 times higher than the normal upper limit and total bilirubin is 3 times higher than the upper limit of normal.
16
Co-morbid systemic illnesses (uncontrolled diabetes, uncontrolled hypertension, cardiac disease, chronic lung disease, chronic kidney disease, immune-suppression and cancer or other severe concurrent disease) which, in the judgment of the investigator, would make the participant inappropriate for entry into this study or interfere significantly with the proper assessment of safety and toxicity of the prescribed treatment.
17
Current participation in another interventional clinical trial (with an investigational drug) that is not an observational registry and have received an investigational intervention 30 days or 5 half-lives (whichever is longer) before the signing the consent.
18
Participation in any other clinical trial of an experimental treatment for COVID-19.
19
Any other clinical/social/ psychiatric condition for which, in the opinion of the investigator, participation would not be in the best interest of the participant (e.g. compromise the well-being) or that could prevent, limit, or confound the protocol-specified assessments.
20
Employee of the investigator or study site, with direct involvement in the proposed study or other studies under the direction of that investigator or study site.

**Method of Generating Random Sequence**	Computer generated randomization
**Method of Concealment**	Not Applicable
**Blinding/Masking**	Open Label

Primary Outcome

Outcome	TimePoints
To compare the efficacy of treatment with COVID-19 Hyper-Immuneglobulin (Human) plus standard of care versus only standard of care in participants with active COVID-19	Mean change from Day 1 to Day 8 in clinical outcome of treatment with COVID-19 Hyper-Immuneglobulin (Human) as compared to the control arm as assessed by 8-point ordinal scale

Secondary Outcome

Outcome	TimePoints
- To assess the efficacy parameters for the treatment with COVID-19 Hyper-Immuneglobulin plus std of care versus only std of care in participants with active COVID-19.<br>- To evaluate the antibody titers of the treatment with COVID-19 Hyper-Immuneglobulin plus std of care versus only std of care in participants with active COVID-19.<br>- To monitor the safety of treatment with COVID-19 Hyper-Immuneglobulin plus std of care versus only std of care in participants with active COVID-19.	- Mean change from Day 1 to Day 3 and Day 14 in clinical outcome of treatment with COVID-19 Hyper-Immuneglobulin (Human) as compared to the control arm as assessed by 8-point ordinal scale<br>- Composite clinical outcome assessed by following up to 14 days<br>- All-cause mortality at day 28<br>- Time to resolution of following symptoms based on 5-point ordinal scale for up to 14 days- Shortness of Breath, Fatigue, Cough, Fever

**Target Sample Size**	**Total Sample Size**="60"<br>**Sample Size from India**="60"<br>**Final Enrollment numbers achieved (Total)**= "0"<br>**Final Enrollment numbers achieved (India)**="0"
**Phase of Trial**	Phase 2
**Date of First Enrollment (India)**	22/09/2020
**Date of Study Completion (India)**	Date Missing
**Date of First Enrollment (Global)**	Date Missing
**Date of Study Completion (Global)**	Date Missing
**Estimated Duration of Trial**	**Years**="0"<br>**Months**="4"<br>**Days**="0"
**Recruitment Status of Trial (Global)** Modification(s)	Not Applicable
**Recruitment Status of Trial (India)**	Completed
**Publication Details**	None (yet)
**Individual Participant Data (IPD) Sharing Statement**	**Will individual participant data (IPD) be shared publicly (including data dictionaries)?**<br><br>**Response** - NO

Brief Summary

Overall Design

This is a prospective, open-label, two-arm, randomized, controlled, multi-centric trial for evaluation of efficacy and safety of COVID-19 Hyper-Immuneglobulin (Human) solution manufactured by Intas Pharmaceuticals Ltd. In participants with active COVID-19.
There will be 2 days of screening period followed by 28 days of study period (including 2 days treatment period with study intervention). Participants will be randomized in 1:1 ratio in treatment arm (T) and control arm [R]. Participant will be randomized EITHER in treatment arm to receive COVID-19 Hyper-Immuneglobulin (Human) solution, 30 mL dose on day 1 & 2 (at the same time preferably) plus standard of care OR in control arm to receive only standard of care.

Brief Summary

The purpose of this study is to compare efficacy and safety of addition of COVID-19 Hyper-Immuneglobulin (Human) solution against standard of care.

Close
