# Supplementary material for: An analysis of deficiencies in the ethics committee data of certain interventional trials registered with the Clinical Trials Registry–India
Source: PLOS Glob Public Health. 2022 Oct 24;2(10):e0000617. doi: 10.1371/journal.pgph.0000617 (PMC10021301; doi:10.1371/journal.pgph.0000617)
Supplement: S3 File — (DOCX) [file pgph.0000617.s003.docx]

**S3 File. Details of 31,423 records from CTRI, stored in an SQLite database**

This database is available at https://osf.io/t3mv5/
