# Supplementary material for: An analysis of deficiencies in the ethics committee data of certain interventional trials registered with the Clinical Trials Registry–India
Source: PLOS Glob Public Health. 2022 Oct 24;2(10):e0000617. doi: 10.1371/journal.pgph.0000617 (PMC10021301; doi:10.1371/journal.pgph.0000617)
Supplement: S5 File — (DOC) [file pgph.0000617.s005.doc]

**S5 file. Expanded methods.**

**1. The R programme to obtain data from CTRI, and store it in an SQLite database.**

We designed an R programme to web scrape data from the records hosted by CTRI. The original data available from CTRI trial webpages was present in a tabular format, where the sub fields sometimes followed a variable pattern. For example, there were cases where all the subtables were missing. This lead to problems while automating the data collection process. The R program looked for trial webpages using the trial IDs as obtained from the URLs for such webpages. This trial ID was not present on the trial record, but was accessed from the relevant trial record URL. Each trial web page was read using the XML package in R that presented the data in the form of a dataframe with 3 to 4 columns. The raw data thus obtained was repeatedly explored to find the required data fields such as *CTRI Number, Type of Trial*, *Phase of Trial*, *No of Ethics Committees,* *No of Sites*, *Name of Committee*, and *Name of Site*. The fields were processed using common R packages such as tidyr, stringr, and readr. The processed data was then stored in a standardised and usable in-house SQLite database.The SQLite database was created and modified using the RSQLite package in R.

**Inconsistencies**

As mentioned earlier, some CTRI records had a different pattern from the rest. We performed extensive string manipulations to handle the data, so that if a particular trial record did not present the data in a standard way, the code handled the issue instead of terminating. For example, CTRI lists the address of the sites of study in a single cell where the name of the ‘place’, ‘city’ and ‘state’, and ‘pincode’ are on consecutive lines. However, the data was not always correctly segmented, and present in these four lines. As we progressed with the project and identified the need to have properly segmented data for this information, the code had to be further modified so that address details could be categorised into the subfields 'place', 'city','state' and 'pincode'.

**Accessing the data from the database and carrying out data related tasks**

The SQLite database contained dedicated tables for each data field, with the relevant CTRI Number and field-related information. This made working with the database easy and simple. The database was accessible using programming languages such as python, R, and so on. It was also accessible using GUIs like DB Browser for SQLite (DB4S). Basic SQL queries were used, as required, to explore and export data from the database. Further analyses were performed using formulae, functions, pivot tables, and so on of Microsoft Excel and LibreOffice Calc spreadsheets software. To use programmes to find the best matches for sites with ECs using python, the input data was exported from the database, and prepared using spreadsheet tools.

A few examples of the basic SQL queries commonly used with the database are as follows:

| **Description** | **Query** |
| --- | --- |
| list all data for Sites of Study | SELECT * FROM sites_of_study; |
| list all data for Ethics Committee | SELECT * FROM ethics_commmittee; |
| list all data related to recruitment for the trials | SELECT * FROM recruitment; |
| list all data for type of trial | SELECT * FROM type_of_trial; |
| list all data related to registration for the trials | SELECT * FROM registration; |
| list all data for phase information of the trials | SELECT * FROM phase; |
| list all data for number of sites and number of ethics committee per trial | SELECT DISTINCT a.trial_id, a.ctri_number, a.noofcom, b.noofsites FROM ethics_committee a JOIN (SELECT DISTINCT trial_id, ctri_number, noofsites FROM sites_of_study)b ON a.trial_id = b.trial_id; |
