# Supplementary material for: An analysis of deficiencies in the ethics committee data of certain interventional trials registered with the Clinical Trials Registry–India
Source: PLOS Glob Public Health. 2022 Oct 24;2(10):e0000617. doi: 10.1371/journal.pgph.0000617 (PMC10021301; doi:10.1371/journal.pgph.0000617)
Supplement: Attachment — Submitted filename: Response to reviewers.doc [file pgph.0000617.s010.doc]

**Response to reviewers, for the manuscript ‘**An analysis of deficiencies in the Ethics Committee data of certain interventional trials registered with the Clinical Trials Registry – India’ by Chakraborty I, et al.

**Note to Editor:**

We have made a few very minor corrections, clarifications, and improvements here and there which are not documented in our response to the reviewers.

**Comments by Reviewer 1, and our responses to them.**

1. “Ethics Committee (EC)” – it might be better to change EC to REC to distinguish research ethics committees from other kinds of ethics committees, such as clinical ethics committees. I recommend that the same change is made in the key words in order to improve the accuracy of key word searches. I do, however, also take note of the following sentence in the discussion: “Even leaving aside a high clinical burden, it is known that ECs may be overburdened with trial-related work”. Does this mean that ECs act as both research ethics committees and clinical committees in India? If so, could this please be described in the Introduction section in order to provide readers with more context of the ethics review system in India?

- The Ethics Committee (EC) or Institutional Ethics Committee (IEC) in India is equivalent to the Research Ethics Committee (REC) or Institutional Review Board (IRB) in other countries. We have explained this in the Introduction now.
- Largely, we do not have Clinical Ethics Committees in India.
- We have added REC and IRB as keywords, but have left the EC/IEC nomenclature as is in the manuscript.
- The following lines in the manuscript are modified versions of the original:

“It is important to have accurate data on which ECs are linked to a given trial that has taken place, or is ongoing, in the country. The EC itself may be over-burdened with trial-related work [23], or physician members of the EC in their individual capacity may have high clinical burdens. Accurate EC data will help determine the frequency with which particular ECs supervise studies, and can quantify the load on each EC, or its physician members, at a given time. It would also be of great assistance in case of any mishap, when public health advocates will be able to immediately identify which EC needs to be contacted, instead of waiting for information from the government or the sponsor. It is known that researchers or health advocates may have to struggle to obtain information in a timely manner, as demonstrated by the Tamiflu case when researchers had a long struggle to obtain the clinical study reports from the sponsor or the regulator [28]. The greater the transparency around the ECs linked to a given trial, the more likely it is that the public will obtain rapid and detailed information in case a trial goes wrong.”

- - - - - - - - - - - - - - - - - - - - - - - - - - - - - - - - - - - - - - - - - -
2. In addition, could the authors provide more information about the range of trials captured on the Clinical Trials Registry–India.

(a) In a CTRI record, the field *Type of trial* has a dropdown option with four options, ie. Observational, Interventional, PMS (post-marketing study) and BA/BE. So, these four categories of trials are held by the registry.

(b) Aside from trials in Western medicine, a large number of trials in ‘alternate’ streams of medicine, ie ayurveda, yoga, unani, siddha and homeopathy (generally known as AYUSH) are conducted in India, which may also be found in CTRI.

These points have been added to the Introduction.

- - - - - - - - - - - - - - - - - - - - - - - - - - - - - - - - - - - - - - - - - -
3. Registries certainly have many uses and the authors identify a number: identifying gaps and errors in registries or between registries; identifying discrepancies between the data in registries and in publications; investigating whether trial results were reported on time”. From this description, it is however not clear what value it has “for the public to determine which committee gave approval to a given study at a given site”. While more justification is provided in the discussion, it would be more informative if a clear rationale could be provided upfront since this is the main focus of the article.

[The justification in the Discussion has been moved up to the Introduction, with some elaboration as described in point 1, above.]

- - - - - - - - - - - - - - - - - - - - - - - - - - - - - - - - - - - - - - - - - -
4. The title refers to “interventional trials”, but the introduction refers to “clinical trials”. The authors state: “we went on to identify the trials of interest in this set”. Could they please explain why they were only interested in interventional trials and not all clinical trials?

[1. We have modified the title to describe the dataset as ‘certain interventional trials’.

2. We felt that Phase 2 and Phase 3 (interventional) trials are the most important trials, since they would include new molecular entities, for instance.

3. A very wide range of trials are registered with CTRI. This includes (a) BA/BE studies, Phase 1 to Phase 4 trials, and post-marketing studies; (b) observational trials; (c) trials related to either modern medicine or traditional systems of Indian medicine; (d) trials that are run as part of postgraduate thesis work (which are very rarely ‘regulatory trials, ie they will usually not be part of submissions to the drug regulator); and (e) trials that may or may not require regulatory clearance to run. To further complicate matters, there are many categories of errors in the CTRI records, some arising from a misunderstanding of what a field ought to capture, as we have documented in our paper (Pillamarapu et al, *Trials*, 2019, <https://doi.org/10.1186/s13063-019-3592-0>). To the extent that different sets of trials may be plagued by different categories of misunderstandings and errors, we felt that limiting the trials to Phase 2 and Phase 3 interventional trials would create a cleaner dataset.]

- - - - - - - - - - - - - - - - - - - - - - - - - - - - - - - - - - - - - - - - - -
5. There were 24,445 trials registered between 1 June 2016 and 19 February 2021, an almost five-year period”. Why was this specific time period selected? Why not rather exactly 5 years?

[We had initially planned to conduct the study for a 5-year period, ie 1 June 2016 to 31 May 2021. It was convenient to start the project on 19 Feb 2021, and we had planned to capture the later records on 1 June 2021. However by that time we forgot about the plan. In fact, we remembered only when we saw this query by the reviewer. In our defence, we will say that although we have ourselves conducted much bigger studies (**Chaturvedi N, et al.** Some data quality issues at ClinicalTrials.gov. Trials 20:378 (2019); https://doi.org/10.1186/s13063-019-3408-2), studies covering three years of trial registry data have also been conducted (Dal-Ré R et al. European non-commercial sponsors showed substantial variation in results reporting to the EU trial registry J. Clin. Epidemiol. 142 (2022) 161–170. https://doi.org/10.1016/j.jclinepi.2021.11.005). So, we request the reviewer to accept this data set.

It is also a cautionary tale for us, to create a mechanism so that we do not forget such plans.]

- - - - - - - - - - - - - - - - - - - - - - - - - - - - - - - - - - - - - - - - - -
6. “17,342 were interventional trials, as identified by the field Type of trial. Of these, 3160 were Phase 2, and 3200 were Phase 3, as identified by the field Phase”. This leaves a large number of trials (a total of 10,982) that were not Phase 2 and/ or 3. Please explain what these trials were.

[We have now undertaken an analysis of the excluded trials, and the statistics are as follows (the details are enclosed in a file for the reviewer’s perusal only).

1. Starting with 17,342 trials, we searched for Phase 2 and Phase 3 trials using the field ‘phase’. Phase 2=3160 trials; Phase 3=3200; Total=6360; Overlap=962; Remaining=5398 trials. 17,342-5398 = **11,944** trials.

2. For the 11,944 trials, the breakup of the data in the Phase field was as follows: Phase 1=1167; Phase 1/Phase 2=675; Phase 4=1223; PMS=422; **N/A= 8457** (It is not clear whether N/A means ‘Not applicable’, or ‘Not available’ – in India N/A is used for either.).

3. The 8457 records were taken forward. We asked whether the words reflecting alternate systems of medicine, ie. ‘ayurveda’, ‘yoga’, ‘unani’, ‘siddha’, ‘homeopathy’, or their commonly used acronym ‘AYUSH’ appear anywhere in the trial record. They did for **2130** records. That left **6327** records.

4. There is a field in the CTRI record that records whether the trial is one that a postgraduate student has conducted for his/her thesis work. **3827** of the 6327 records were ‘PG theses’ = ‘yes’. **2495** were ‘no’ and **5** were blank. Therefore, 2495 + 5 = **2500** records were ‘not yes’ for PG thesis.

5. There is a field in the CTRI record entitled ‘Regulatory Clearance Status from DCGI’ (ie from the Drugs Controller General of India). We checked this field for the 2500 records. It was ‘Not applicable’ for **2447** trials, meaning that these were non regulatory trials. This left 53 trials.

6. Of the 53 trials, only 24 were ‘closed to recruitment’ or ‘completed’. The remaining 29 were ‘Not Yet Recruiting’, ‘Open to Recruitment’ or ‘Other (Terminated)’.

In summary, we excluded trials that were not ‘Phase 2 or Phase 3 trials and either Completed or Closed to recruitment’. Some of these trials were (i) Phase 1, Phase 1/Phase 2, Phase 4 or post-marketing studies, (ii) part of alternate systems of medicine, (iii) studies as part of postgraduate thesis work; (iv) studies that did not require regulatory clearance to run; and (v) not ‘closed to recruitment’ or ‘completed’. 24 other trials did not fit any of these criteria, but did not list Phase 2 or Phase 3 in the phase field.]

- - - - - - - - - - - - - - - - - - - - - - - - - - - - - - - - - - - - - - - - - -
7. Why were Phase 1 trials excluded?

In a CTRI record, the field *Type of trial* has a dropdown option with four options, ie. Observational, Interventional, PMS and BA/BE. In earlier work (Pillamarapu et al, *Trials*, 2019, https://doi.org/10.1186/s13063-019-3592-0) we found that BA/BE are sometimes confused with Phase 1 studies (ie *Type of trial* is BA/BE, but *Phase* is Phase 1). Given the large generics’ industry in India, many ‘Phase 1’ trials are likely to be BA/BE studies. In order to keep the dataset as well defined as possible, we limited the study to Phase 2 and Phase 3 trials.

- - - - - - - - - - - - - - - - - - - - - - - - - - - - - - - - - - - - - - - - - -
8. A total of 1576 trials were identified but only 1359 were used. Were all the others duplicates? If so, why would there be such a large number of duplicates in the registry?

[The breakup of the 1576 trials was as follows:

Phase 2

Phase 2:	519

Phase 2/3:	217

Total:	736

Phase 3

Phase 2/3:	217

Phase 3:	499

Phase 3/4:	124

Total:	840

The duplicates were the 217 Phase 2/3 trials. This duplication was not an error – it was simply the way our filters worked. We corrected for it by getting rid of one set of 217 Phase 2/3 trials.

We have clarified the nature of the duplicates in the manuscript now.]

- - - - - - - - - - - - - - - - - - - - - - - - - - - - - - - - - - - - - - - - - -
9. “For the unique set of 1359 trials, we went on to identify all those for which (i) no EC was listed; (ii) the number of sites exceeded the number of ECs; (iii) the number of ECs exceeded the number of sites; and (iv) the number of sites was the same as the number of ECs.” For readers to make sense of this analysis, can the authors please briefly explain the ethics review system in India. For instance, does each research site have its own research ethics committee? Are some research ethics committees able to approve more than one research site? Are academic researchers expected to obtain additional ethics approval from the research ethics committee of their own institution if the research site is not affiliated to their institution? While some such relevant information is provided in line 320 onwards, it would be helpful for readers to have more context upfront.

[We have moved the paragraph from line 320 onwards up. We have also added explanatory notes. The resulting paragraphs are as follows:

**In the Introduction:**

... Also, typically institutions have their own EC, and researchers are expected to get approval of their own institution's EC to participate in a trial. It is possible to have multicentre review, where the EC of one site has oversight of all the trial sites, but that would be allowed only for low-risk, non-regulatory trials, ie not the phase 2 and phase 3 trials of this study. We also need to be aware of the regulatory updates of the Government of India. In March 2019, the Ministry of Health and Family Welfare issued the New Drugs and Clinical Trials Rules [21]. These rules permitted a trial to be conducted at a site that lacked its own EC. In such cases, an EC from another institution, or an independent EC, may review the trial provided the EC is located in the same city as the site or is within a 50 km radius. These rules came into effect on 25 March 2019.

**In the Discussion:**

In the 53 trials that had more sites than ECs, we found that the two studies that were registered prospectively on or after 25 March 2019 were compliant with the rule that required the EC to be located within the same city as, or within 50 km of, the trial site. If the EC is able to discharge its duties better with such restrictions on its location, then the value of this aspect of the New Drugs and Clinical Trials Rules, should have impact in trials registered after the rule took effect.]

- - - - - - - - - - - - - - - - - - - - - - - - - - - - - - - - - - - - - - - - - -
10. “For many of the 1359 trials, EC names were listed in a sufficiently detailed manner.” Please quantify “many”.

[We are unable to quantify “many” for the following reason: A given institution is permitted to have multiple ECs, and bigger hospitals tend to do so. For instance, the King Edward Memorial (KEM) Hospital, Mumbai has three ECs, as mentioned in the following news item: <https://science.thewire.in/health/how-do-you-catch-mistakes-in-a-clinical-trial-an-ethics-expert-explains/>. In our study, we encountered (a) Institutional Ethics Committee (IEC-I) and (IEC-II) were involved in one of the sites of the trial CTRI/2017/08/009485 and (b) IEC-I,II,III- TATA Memorial Hospital were involved in one of the sites of the trial CTRI/2019/06/019509. (To be noted: In each case, this listing of multiple ECs was in a single cell, so it did not complicate the issue of how many ECs approve a given site when one tries to match the ‘EC cell’ with the ‘site cell’.) Therefore, a major issue relates to whether a given institution has only one EC. If so, then when ‘EC’ or ‘IEC’, in full form or as an acronym, is listed, it is unambiguously the single committee. Undoubtedly in many cases it is so. But there is no easy way to determine whether, indeed, the institution has only one EC. Therefore we have not considered the use of the terms ‘EC’ or ‘IEC’ as a definitive identification of a unique EC.

Accordingly, we have deleted the following sentences: “For many of the 1359 trials, EC names were listed in a sufficiently detailed manner. However, for others they were not.” We have also summarized the explanation above in the discussion.]

- - - - - - - - - - - - - - - - - - - - - - - - - - - - - - - - - - - - - - - - - -
11. “(ii) Generic names of the EC were used quite often. The name of the institution may, or may not, have been listed in this field as well. (iii) Some entries appeared to be casual references to particular ECs, rather than their proper names.” What is the difference between “generic names” and “casual references”?

[An example of a generic name is ‘institutional ethics committee’. Many institutions use this, although it may not be the formal name of the ethics committee. An example of a casual name is ‘ethical committee of Council’ where it is unclear which Council, and it is unlikely that the committee is named ‘ethical committee’. We have now provided these examples in the manuscript.]

- - - - - - - - - - - - - - - - - - - - - - - - - - - - - - - - - - - - - - - - - -
12. Table 5: “To be noted, ‘trial’ means ‘trial record’” should not be part of the table title but should rather be added as a footnote.

[This has been corrected]

- - - - - - - - - - - - - - - - - - - - - - - - - - - - - - - - - - - - - - - - - -
13. It is unclear how 5 and 6 are different:
5. “A trial with one generically named ‘Institutional Ethics Committee’ for multiple sites.
6 A trial in which a generically named ‘Institutional Ethics Committee’ was listed multiple times.

[In point 5, only one ‘Institutional Ethics Committee’ is listed. But there are 4 sites, in very different parts of the country.

In point 6, there are 4 sites and 4 ECs. 3 of these 4 ECs are called ‘Institutional Ethics Committee’.

These clarifications have been provided in Table 5 now.]

- - - - - - - - - - - - - - - - - - - - - - - - - - - - - - - - - - - - - - - - - -
14. Can the authors please explain what they mean by the following: “Although clarification should not be necessary, this study has found that many trials had errors or ambiguities in their listed ECs.” Why would clarification not be necessary?

[The phrase 'Although clarification should not be necessary' is itself unnecessary. We have removed it.]

**- - - - - - - - - - - - - - - - - - - - - - - - - - - - - - - - - - - - - - - - - -**
15. “Some of those that were registered before this date were not, which highlights the importance of such a rule.” It is not clear to me why trials registered before the rule came into effect should have been compliant with the rule.

[We have reworded this sentence.]

- - - - - - - - - - - - - - - - - - - - - - - - - - - - - - - - - - - - - - - - - -
16. “It is possible that after the trial begins, the EC renews its registration and receives a new number.” Would it not be sensible to recommend that research ethics committee registration numbers remain the same but that the year of registration is indicated?

[We have now recommended the reviewer’s suggestion.]

- - - - - - - - - - - - - - - - - - - - - - - - - - - - - - - - - - - - - - - - - -
17. “Even leaving aside a high clinical burden, it is known that ECs may be overburdened with trial-related work”. Does this mean that ECs act as both research ethics committees and clinical committees?

[Please see the response to point 1, above.]

- - - - - - - - - - - - - - - - - - - - - - - - - - - - - - - - - - - - - - - - - -
18. “Based on a range of parameters, a recent study ranked CTRI eleventh out of the 18 major public trial registries”. Please clarify where these registries are located.

[WHO recognizes 17 registries as Primary Registries. They are located all over the world, including China, Australia, South Korea, India, Sri Lanka, Africa (the Pan African registry), Europe (the EU registry), Brazil and Peru. Although it does not recognize the US’s ClinicalTrials.gov as a Primary Registry, it calls this registry a ‘data provider’ (to its platform, which one can use to search trials in all 18 registries). The other 17 are also called data providers. The reason WHO cannot ignore the US’s registry is because this registry is by far the biggest, with over 414,000 records today, and is several times bigger than the next registry.

The manuscript has been modified, and the sentence now reads:

“Based on a range of parameters, a recent study ranked CTRI eleventh out of the 18 major public trial registries that included ClinicalTrials.gov of the United States and the 17 registries that WHO considers Primary Registries [1].”]

- - - - - - - - - - - - - - - - - - - - - - - - - - - - - - - - - - - - - - - - - -
Small issues:
19. “registry data has” - the word data is plural.

[This has been corrected, in this phrase and elsewhere.]

- - - - - - - - - - - - - - - - - - - - - - - - - - - - - - - - - - - - - - - - - -
20. “the trials enterprise” – should this be the clinical trials enterprise?

[This has been corrected.]

- - - - - - - - - - - - - - - - - - - - - - - - - - - - - - - - - - - - - - - - - -
21. “Repeatedly, academics and others, including the Science and Technology Committee of the UK House of Commons [3–5]…”. Since only reference 5 refers to the Science and Technology Committee of the UK House of Commons, it would be better to place the references as follows: Repeatedly, academics and others [3,4], including the Science and Technology Committee of the UK House of Commons [5]...

[This has been corrected.]

- - - - - - - - - - - - - - - - - - - - - - - - - - - - - - - - - - - - - - - - - -

**Comments by Reviewer 2, and our responses to them.**

Reviewer #2: Detailed Comments attached. This research has been conducted without any ethics committee approval or without seeking an exemption from ethics committee before undertaking this analysis or without registering the study on CTRI platform before its conduct. Further this paper is based on an interpretation of findings and not on verified facts/ discussion regarding data in consultation with data managers. This is not a true presentation of facts and not recommended for publication in this esteemed journal.

[a. As stated in many papers, research involving publicly available data does not require EC approval or exemption from an EC. Two examples are our own papers, both from 2019, <https://doi.org/10.1186/s13063-019-3592-0> and <https://doi.org/10.1186/s13063-019-3408-2>. Others’ studies that state that EC approval is not required for studies of this type include doi 10.34172/ijhpm.2021.172, at <https://www.ijhpm.com/article_4180_f5d9c6aa68a33126c9b5d4538deb5086.pdf> (2021) and <http://dx.doi.org/10.1016/j.jclinepi.2014.06.019>, at <https://www.jclinepi.com/article/S0895-4356(14)00276-5/fulltext> (2015).

b. Only clinical studies are registered on CTRI, not research about data pertaining to clinical studies.

c. We have been extremely transparent about every step of our analysis, with voluminous data in the supplementary files. Everything is verifiable.

d. We did not discuss the study with the CTRI managers, and have addressed this point in 5(i) below.]

- - - - - - - - - - - - - - - - - - - - - - - - - - - - - - - - - - - - - - - - - -

**Comments on An analysis of deficiencies in the Ethics Committee data of interventional trials registered with the Clinical Trials Registry – India**

The manuscript has examined the data on the Ethics Committee (EC) field for trials registered in Clinical Trial Registry of India (CTRI). Comments are as below;

1. Ethics Review**–** The study has not followed ICMR National Ethical Guidelines, 2017. An appropriate Ethics review review would have ensured that only robust scientific methods and tools are employed for extracting data and analysis so that the study does not draw conclusions on limited data and skewed perceptions. <https://main.icmr.nic.in/sites/default/files/guidelines/ICMR_Ethical_Guidelines_2017.pdf>.

[The corresponding author has used similar methodology in an earlier paper (Pillamarapu et al, *Trials*, 2019, <https://doi.org/10.1186/s13063-019-3592-0>). Broadly speaking, other researchers have also filtered registry data in a manner similar to what we have done to pose various questions of such registries (<https://doi.org/10.1371/journal.pone.0226143> (2020), DOI: 10.1002/jrsm.1500 (2021) and <https://doi.org/10.1186/s13063-021-05423-1> (2021).

The reviewer has not pin pointed any specific problems with our methodology, except to make a general statement. We stand by our methodology, which is laid out in detail, for reproducibility.]

- - - - - - - - - - - - - - - - - - - - - - - - - - - - - - - - - - - - - - - - - -

2. CTRI Registration- This study was not found to be registered on the CTRI platform. This seems to have been overlooked as all research studies should be registered.

[CTRI is a platform to register clinical studies – observational, interventional, post-marketing studies and BA/BE studies. It is not a platform to register studies of the sort that this manuscript reflects, that is an analysis of the trials that are registered with CTRI.]

- - - - - - - - - - - - - - - - - - - - - - - - - - - - - - - - - - - - - - - - - -

3. Objectives & Design- The study does not seem to have any scientific research questions that it is trying to answer. It doesn’t state the aim or any primary and secondary endpoints, therefore the conclusion drawn from the study are inappropriate. The study has limited value in the advancement of scientific knowledge

[Our work is policy-research, not science. Also, it is not a clinical trial, and therefore does not have primary or secondary endpoints. In terms of objectives:

a) In the Abstract we state ‘In this study, we examined the trial sites and ethics committee (EC) data for 1359 recent Phase 2 or Phase 3 interventional trials registered with Clinical Trials Registry– India (CTRI), to identify categories of problems that prevent the clear identification of which EC approved a given site.’

b) And in the Introduction, Line 85 we state ‘...we wished to perform a more detailed audit of the EC data in CTRI and identify any other lacunae with this data.’]

- - - - - - - - - - - - - - - - - - - - - - - - - - - - - - - - - - - - - - - - - -

4. Choice of Database-

(i) All clinical trials must be registered with the Clinical Trial Registry -India (CTRI). While such data can provide quick and easy access to information for clinical trial administrative information for improving transparency and accountability, it may be noted that the authors have not analysed any ‘Clinical trial information’ but focussed on ‘ethics committees’ related field without highlighting the fact that this is not an Ethics Committee Registry.

[The EC is a crucial part of a trial, and as a primary registry of the WHO, CTRI is a valid source for trial-related EC information. In this work, we examined the ECs from the view point of the trials that each of them were involved in. We believe that readers should be able to understand this.]

- - - - - - - - - - - - - - - - - - - - - - - - - - - - - - - - - - - - - - - - - -

(ii) This is a wrong choice of database to analyse EC related details and further information available on SUGAM Portal which carries the CDSCO Registry of ethics committees <https://cdsco.gov.in/opencms/opencms/en/Clinical-Trial/Ethics-Committee/Ethics-Committee-Registration/> or on NAITIK Portal with the DHR National Registry of Ethics committees reviewing biomedical and health research in India. <https://naitik.gov.in/DHR/Homepage> has not at all been referred during analysis. Even the discussions do not mention about these databases

[If we were analyzing ECs per se, without reference to the trials that they are involved in, then we would certainly have to look at the data available with the SUGAM and NAITIK portals. Since our work focuses on the EC-trial site linkages, and the identification of the ECs in that context, we belive that it is not necessary to refer to SUGAM and NAITIK.]

- - - - - - - - - - - - - - - - - - - - - - - - - - - - - - - - - - - - - - - - - -

5. **Methodology and results** –

(i) It may be noted that Detailed Audits cannot be conducted like this as stated by the Authors on line 85 to identify lacunae with this data. Due collaboration/ partnership is required from where data is sourced for undertaking meaningful analysis and making relevant observations which was not done.

[On three separate occasions, the PI has written to, or otherwise attempted to contact, Dr. VishnuVardhan Rao, who heads the CTRI team, seeking a collaboration. There has never been a response. Whereas it is true that a partnership may enable us to access data that is not in the public domain, there are two problems with this approach: (a) the records are meant for the public, and should stand on their own, with high quality data; and (b) conceivably, a collaboration could muzzle us from publishing accounts that are critical of CTRI data. We have reason to believe that this might have happened with another study.]

- - - - - - - - - - - - - - - - - - - - - - - - - - - - - - - - - - - - - - - - - -

(ii) There is no information regarding validation of the tool developed in-house to extract data and undertake analysis. Unless the data processing is appropriate and it is difficult to undertake meaningful analysis.

[All data has been handled by the very detailed Methodology that is described in the manuscript. Two authors have independently performed each step of the Methodology. We stand by our processes.]

- - - - - - - - - - - - - - - - - - - - - - - - - - - - - - - - - - - - - - - - - -

(iii) On line 125 it is mentioned that many EC names were listed in sufficiently detailed manner however corresponding numbers are not provided.

[We are unable to quantify “many” for the following reason: A given institution is permitted to have multiple ECs, and bigger hospitals tend to do so. For instance, the King Edward Memorial (KEM) Hospital, Mumbai has three ECs, as mentioned in the following news item: <https://science.thewire.in/health/how-do-you-catch-mistakes-in-a-clinical-trial-an-ethics-expert-explains/>. In our study, we encountered (a) Institutional Ethics Committee (IEC-I) and (IEC-II) were involved in one of the sites of the trial CTRI/2017/08/009485 and (b) IEC-I,II,III- TATA Memorial Hospital were involved in one of the sites of the trial CTRI/2019/06/019509. (To be noted: In each case, this listing of multiple ECs was in a single cell, so it did not complicate the issue of how many ECs approve a given site when one tries to match the ‘EC cell’ with the ‘site cell’.) Therefore, a major issue relates to whether a given institution has only one EC. If so, then when ‘EC’ or ‘IEC’, in full form or as an acronym, is listed, it is unambiguously the single committee. Undoubtedly in many cases it is so. But there is no easy way to determine whether, indeed, the institution has only one EC. Therefore we have not considered the use of the terms ‘EC’ or ‘IEC’ as a definitive identification of a unique EC.

Therefore, the following sentence has been deleted: “For many of the 1359 trials, EC names were listed in a sufficiently detailed manner. However, for others they were not.”. We have also summarized the explanation above in the discussion.]

- - - - - - - - - - - - - - - - - - - - - - - - - - - - - - - - - - - - - - - - - -

(iv) On 140 authors have reported 53 trials listing more sites than ECs and listed this as a problem. This shows a lack of understanding as authors want that number of sites vs ethics committees to be an exact match, whereas, in cases, such as, if common review of multicentre research is done, joint EC reviews, or site without an EC has approached neighbouring site EC or an independent EC for review and other situations the case is likewise. Similarly on 150, 38 sites have listed more ECs and without verification with data managers, it is unclear how authors have listed this as a problem and made several interpretations of what might be the reasons.

[We have now changed line 140 from ‘These studies had the following problems with their site and EC data:’ to ‘These studies had the following actual or possible problems with their site and EC data:’. For lines 150–166, we by-and-large stand by our original statements. However, here too, we have added the phrase “These studies had the following actual or possible problems with their site and EC data”.

However, for lines 167–170 we have modified the write up, which is now as follows: .

i) We first consider the listing of more ECs than sites. The CTRI staff have explained one possible cause of this [29], as follows. If new sites are to be added to the CTRI record, the trialist first uploads the ethics approval documents. Then the site field is unlocked and the new site information is added. There may be a lag between the two steps, leading to an excess of ECs over sites for the intervening period. We suggest that (a) the public should not see each step of the process, and only get to see the final changes, or (b) there should be some indication that updating the record with new EC and site information is ongoing. Another possible reason for more ECs is that sites may have been dropped, without the corresponding changes in the list of ECs. Other situations may be as follows. For particular trials, one EC may seek the opinion of another EC before approving a trial. Conceivably, this may lead to more than one EC per site being listed for that trial. Also, in ‘multicentre review’ the EC of one institution may approve and oversee the trial at all the sites, with the local EC also involved in overseeing the trial. However this would happen in low risk non-regulatory trials, and would not be possible in the Phase 2 and Phase 3 regulatory trials of this study.”

We disagree that we have found fault with anything but a ‘one site, one EC’ scenario. We have discussed the issue of the possibility of one EC handling more than one site, and the New Drugs and Clinical Trials Rules of March 2019 which restricts such an EC to the same city or a 50 km radius of the site. We have also mentioned independent ECs.

Regarding verifying data with the CTRI managers, please see our response 5(i)]

- - - - - - - - - - - - - - - - - - - - - - - - - - - - - - - - - - - - - - - - - -

(v) It may be further seen that out of 1359 trials 1265 trials has equal numbers of EC and Sites but this has not been discussed.

[We disagree. The 1265 trials with equal numbers of EC and sites were broken up into three sets, containing 987, 236 and 42 trials (Table 3). The set of 236 trials have been discussed explicitly. Almost all of the various problems for the sets of 987 and 42 trials have been discussed without explicit reference to these numbers.]

- - - - - - - - - - - - - - - - - - - - - - - - - - - - - - - - - - - - - - - - - -

(vi) The paper provides no clear inclusion and exclusion criteria, pl see 100-110 discussing ‘interventional trials’ without any clear definition. While on 260 the analysis discusses about focussing on ‘regulatory trials’. These terms cannot be equated and CTRI database registers all types of clinical as well as biomedical research studies. Table 1. Is unclear and unable to explain why something is getting included or excluded from this analysis.

[We believe that our inclusion and exclusion criteria have been very clearly stated. Line 104 says “17,342 were interventional trials, as identified by the field *Type of trial.”* In a CTRI record, the field *Type of trial* has a dropdown option with four options, ie. Observational, Interventional, PMS and BA/BE. For a recent period of several years, we chose the trials that had ‘Interventional’ in the *Type of trial* field, and this amounted to 17,342 trials.

Line 264 refers to ‘regulatory trials’. These are the most important trials, because they need to be cleared by the drugs regulator, that is the Central Drugs Standard Control Organisation (CDSCO), before they can run (Pandey et al. Pharm Med. 2013; 27:83–93. https://doi.org/10.1007/s40290-013-0009-3). All phase 2 and phase 3 trials would have to be so approved. So, ‘regulatory trials’ are a subset of ‘interventional trials’. Nevertheless, we have modified the wording and removed ‘regulatory trials’.

We believe that Table 1 is self-explanatory. In response to Reviewer 1’s comments, we have provided slightly more detail in the paragraph above Table 1.]

- - - - - - - - - - - - - - - - - - - - - - - - - - - - - - - - - - - - - - - - - -

(vii) On 290 authors make contradictory remarks, say there is need for intensive quality checks, and in the same line say it is happening already but needs to be strengthened.

[We write “There also needs to be a more intensive quality check of a submission prior to it being allowed to be published. Although automated checks would be the most efficient, it is essential that a CTRI staffer review the record in a detailed manner, to minimize errors and ensure that the data is meaningful. Although this is happening already [20], it clearly needs to be strengthened.” We do not believe that this is contradictory. A process to check for quality exists, but clearly some issues are being missed in the quality check, and therefore the process needs to be strengthened.]

- - - - - - - - - - - - - - - - - - - - - - - - - - - - - - - - - - - - - - - - - -

(viii) On 445 authors talk about need for training of EC, however this observation has no connection to this analysis or to quality of data that has been entered in CTRI records by Sponsors / Investigators.

[As part of the Discussion, we state “Over the years, researchers have pointed out the many steps that need to be taken to improve the ethics of clinical trials in India, including more training of EC members [26], increased active monitoring of ongoing trials [21], and the need for more ECs to be accredited [26], including by international organizations [21]. Our focus has been the quality of CTRI records.” Yes, the point is not directly related to this study, but we believe that it is fine as part of the Discussion. We make no attempt to conflate the point about EC training with the data in our study, or our interpretation thereof.]

- - - - - - - - - - - - - - - - - - - - - - - - - - - - - - - - - - - - - - - - - -

6. Missing references for International Databases-

(i) Authors have correctly noted that when the Clinical Trial Registry of India was launched in the year 2007, even the WHO Clinical Trial Registry had 20 point did not seek any EC related information. And thus India was probably the first in the world to include EC related information in its clinical trial database.

(ii) But the authors have failed to inform or discuss in the paper, that even the largest International public database of Clinical Trials namely the Clinical Trial.gov, only asks for EC approval status and does not require any identified EC related information (conditionally required), and even if EC information is collected for admin purposes, it is not displayed on public website and this includes information such as ethics committees name, approval number, or contact information. <https://prsinfo.clinicaltrials.gov/definitions.html>

[As the reviewer notes in the point before this, India can be rightly proud that it listed EC-related information even before WHO required it. Nevertheless, it there is a field, it needs to be correctly filled. Otherwise, the field should be removed. There is no point being proud of something that happened in 2007, if it does not serve the required purpose in 2022.]

- - - - - - - - - - - - - - - - - - - - - - - - - - - - - - - - - - - - - - - - - -

7. References: The authors have self quoted themselves to establish their own theory of finding various other uses of clinical trial registries such as at SN 1, 2, 9, 26. This seems to be an attempt to establish multiple uses of Clinical Trial Database uses, as per their assumption.

[We have built on our earlier work, and have self cited where relevant. All researchers do this. Other researchers have also cited our earlier papers.]

- - - - - - - - - - - - - - - - - - - - - - - - - - - - - - - - - - - - - - - - - -

8. Manuscript-

(i) Most of the problems stated by Authors, cannot be at all be called ‘deficiencies’ of the EC data but just a different way of listing names of institutions or names of EC.

[We disagree. If an EC cannot be identified unambiguously, and an EC-site linkage cannot be clearly identified, then what is the point of listing the EC data? The ‘deficiencies’ of the EC data that we point out are real. In many cases, the data entered in the EC field is so poor that it is equivalent to not being entered.]

- - - - - - - - - - - - - - - - - - - - - - - - - - - - - - - - - - - - - - - - - -

(ii) The paper has completely failed to recognise that institutions could have other arrangements, and label them as problems or deficiencies, such as joint EC arrangements, or have more than one EC in an institution or approach an independent EC for review and so on. Authors seem to have only focussed on one institution one corresponding EC theory.

[We have described a range of problems with the EC data. However we have addressed the reviewer’s concern about the ‘one institution one corresponding EC theory’ in point 5(iv), above.]

- - - - - - - - - - - - - - - - - - - - - - - - - - - - - - - - - - - - - - - - - -

(iii) The authors have elaborated heavily on trivial matters, related to filling of forms, and prepared a full paper focussing on types of errors in typing, misspellings, acronyms, and calling for better transparency and labelling these as deficiencies.

[We have clearly stated the rationale of our study, in point 3 above. Dr. Deborah Zain, who headed ClinicalTrials.gov for many years has also pointed out the many trivial errors that were found in that registry at the time of their work, in DOI: 10.1056/NEJMsr1601330 (2017).

On a slightly different note, we would refer the reviewer to a recent report by Prof. Ben Goldacre (Goldacre, B & Morley, J. (2022). Better, Broader, Safer: Using health data for research and analysis. A review commissioned by the Secretary of State for Health and Social Care. Department of Health and Social Care.). This is a document, magesterial in its sweep, that is preparing the clinical trial reporting enterprise in the UK for the next several decades. It should be our collective goal to make CTRI one of the best registries in the world, fit for purpose for many years to come. We should not be defensive about the current deficiencies in the trial records, and instead learn from them, to make future records as free of mistakes as possible.]

- - - - - - - - - - - - - - - - - - - - - - - - - - - - - - - - - - - - - - - - - -

(iv) Poorly written script, haphazard writeup, confused approach, lengthy tables, completely lacks clarity, focus and quality, written with skewed understanding and own interpretations, drawing far-fetched conclusions without data, indicates lack of understanding on the subject, and an effort to analyse things beyond the scope of CTRI database.

[As pointed out by both reviewers, there were some deficiencies in the manuscript, which we have attempted to correct. However we disagree with this wholesale condemnation.]

- - - - - - - - - - - - - - - - - - - - - - - - - - - - - - - - - - - - - - - - - -

(v) The conclusions have no relevance, as no efforts were made to undertake this in consultation with relevant stakeholders and therefore lacking key consultations/ confirmations to base their recommendations upon.

[Please see response to query 5(i), above.]

- - - - - - - - - - - - - - - - - - - - - - - - - - - - - - - - - - - - - - - - - -

9. Misleading will increase reluctance to register CT –

(i) This publication looks like a fault finding exercise, based on incomplete data will do more societal harm by misinforming and further causing doubts in minds to its purpose and quality of CTRI which may thereby increase in hesitation /reluctance to register among researchers to register Clinical trials in the database.

[Many users of CTRI are already very critical of the quality of its records (personal communications from a few people to the corresponding author). This publication is unlikely to cause doubts in the minds of users. Rather, it may relieve them that a documentation of certain categories of problems has been done, which may lead to an improvement in the registry.

On a separate note, researchers are legally required to register certain categories of trials, so there is no question of reluctance to register based on the information in this manuscript.]

- - - - - - - - - - - - - - - - - - - - - - - - - - - - - - - - - - - - - - - - - -

(ii) Publications with biased viewpoints are severely detrimental to the public health goals. CTRI is well recognised by WHO as one of the Primary Registries in the WHO Network of Registries to fulfil requirements. <https://www.who.int/clinical-trials-registry-platform/network/primary-registries>.

[CTRI is a primary registry of the WHO. So is the Dutch registry, the Netherlands Trial Register (NTR). In our paper (Venugopal and Saberwal, PLOS ONE, 2021, https://doi.org/10.1371/journal.pone.0251191), in which we analyzed 18 registries (17 WHO-recognized registries and ClinicalTrials.gov) on 14 criteria, we were highly critical of NTR, and ranked it at the bottom of the 18 registries. Recently NTR seems to have closed down. Its website <https://www.trialregister.nl/> says “The Netherlands Trial Register is now permanently closed for new study registrations. We advise you to register your study in another WHO recognised primary registry.”. Therefore, being recognized by WHO doesn’t seem to be a mark of quality for trial registries.]

- - - - - - - - - - - - - - - - - - - - - - - - - - - - - - - - - - - - - - - - - -

The paper does not advance any science or knowledge, its methodology is biased, inclusion exclusion criteria are unclear, have an unscientific basis and conducted without an EC approval. In view of the above observations, this is not recommended for publication in this esteemed journal.

[Our work is policy-research, not science. This paper is part of the genre that seeks greater transparency and accountability of trial registries. A leading light in this kind of work is Prof. Ben Goldacre of the University of Oxford. He has given testimony to the Science and Technology Committee of the UK House of Commons. Clearly, the UK government sees great value in this kind of work, and we, too, believe in it.]

- - - - - - - - - - - - - - - - - - - - - - - - - - - - - - - - - - - - - - - - - -
