# Supplementary material for: An analysis of deficiencies in the ethics committee data of certain interventional trials registered with the Clinical Trials Registry–India
Source: PLOS Glob Public Health. 2022 Oct 24;2(10):e0000617. doi: 10.1371/journal.pgph.0000617 (PMC10021301; doi:10.1371/journal.pgph.0000617)
Supplement: Attachment — Submitted filename: Response to reviewers.doc [file pgph.0000617.s011.doc]

**Manuscript:** ‘An analysis of deficiencies in the Ethics Committee data of certain interventional trials registered with the Clinical Trials Registry – India’ by Chakraborty I, et al.

**Comments by Reviewer #3, and our responses to them.**

1) a brief description of the steps required by local regulations for the registration and ethical evaluation of projects until their approval for initiation are described;

The following lines have been added to the Introduction:

Here, we briefly outline the steps typically required by local regulations for the registration and ethical assessment of projects until their approval are:

a) Typically, projects need to be carried out with institutional support.

b) After a technical assessment (which could also be part of the ethics review process in some institutions) projects are submitted to the institutional ethics committee of the relevant institution(s) for approval.

c) Clinical trial projects also need to be registered with clinical trial registries, especially the Clinical Trials Registry – India, prior to recruitment of the first participant into the study.

d) For regulatory studies, permission is also needed from CDSCO.

e) For collaborative research involving international partners, permission is required from the Health Ministry Screening Committee.

f) For specific kinds of studies, additional permissions might be required - for example, for gene therapy research, approval from the Gene Therapy Advisory and Evaluation Committee would be required; for samples being shipped to other institutions, Material Transfer Agreements should be in place and special permissions might be required for export or import of samples; for studies involving handling of microorganisms or genetically engineered organisms, permissions from Institutional Biosafety Committees and other relevant bodies such as Review Committee on Genetic Manipulation might be required.

- - - - - - - - - - - - - - - - - - - - - - - - - - - - - - - - - - - - - - - - - -
2) the inclusion of the meaning of the acronym "BA/BE studies".

This has been done: BA/BE studies (ie, Bioavailability/Bioequivalence studies)

- - - - - - - - - - - - - - - - - - - - - - - - - - - - - - - - - - - - - - - - - -
